# POSS-Derived Synthesis and Full Life Structural Analysis of Si@C as Anode Material in Lithium Ion Battery

**DOI:** 10.3390/polym11040576

**Published:** 2019-03-29

**Authors:** Ziyu Bai, Wenmao Tu, Junke Zhu, Junsheng Li, Zhao Deng, Danpeng Li, Haolin Tang

**Affiliations:** 1 State Key Laboratory of Advanced Technology for Materials Synthesis and Processing, Wuhan University of Technology, Wuhan 430070, China; 18971142560@163.com (Z.B.); bebetterzjk@163.com (J.Z.); dengzhao@whut.edu.cn (Z.D.); 18435138790@163.com (D.L.); 2 School of Chemistry, Chemical Engineering and Life Sciences, Wuhan University of Technology, Wuhan 430070, China

**Keywords:** Octavinyl-POSS, organic-inorganic crosslinking, Si@C anode, lithium ion battery, mechanism analysis

## Abstract

Polyhedral oligomeric silsesquioxane (POSS)-derived Si@C anode material is prepared by the copolymerization of octavinyl-polyhedral oligomeric silsesquioxane (octavinyl-POSS) and styrene. Octavinyl-polyhedral oligomeric silsesquioxane has an inorganic core (-Si_8_O_12_) and an organic vinyl shell. Carbonization of the core-shell structured organic-inorganic hybrid precursor results in the formation of carbon protected Si-based anode material applicable for lithium ion battery. The initial discharge capacity of the battery based on the as-obtained Si@C material Si reaches 1500 mAh g^−1^. After 550 charge-discharge cycles, a high capacity of 1430 mAh g^−1^ was maintained. A combined XRD, XPS and TEM analysis was performed to investigate the variation of the discharge performance during the cycling experiments. The results show that the decrease in discharge capacity in the first few cycles is related to the formation of solid electrolyte interphase (SEI). The subsequent rise in the capacity can be ascribed to the gradual morphology evolution of the anode material and the loss of capacity after long-term cycles is due to the structural pulverization of silicon within the electrode. Our results not only show the high potential of the novel electrode material but also provide insight into the dynamic features of the material during battery cycling, which is useful for the future design of high-performance electrode material.

## 1. Introduction

Owing to the aggravation of energy crisis, the demand for new energy conversion and storage devices is growing continuously in recent years. As a promising chemical power supply for electronic device and electric vehicles [1,2], the lithium ion battery has found enormous applications in a variety of applications due to its advantages of high energy storage density and high open circuit voltage [3,4]. At present, graphite carbon is used as negative electrode in the commercialized lithium ion battery system. The theoretical capacity of graphite is only 372 mAh g^−1^ and replacing graphite in the anode with a robust material of high capacity may lead to a battery with higher energy density [5,6,7,8,9]. The theoretical specific capacity of silicon is up to 4200 mAh g^−1^, one order of magnitude higher than that of graphite anode material. Furthermore, the insertion/deinsertion potential of lithium ion for silicon is moderate [10,11]. Due to these advantages, a silicon-based material is an ideal choice for the next generation of lithium ion battery anode. However, during the alloying reaction of silicon with lithium, silicon material will suffer severe volume expansion, which can easily lead to rapid pulverization and detachment of the active material from current collector, which causes the rapid decline of the battery performance [12]. Meanwhile, the silicon material cannot form stable solid electrolyte interface (SEI). The newly exposed silicon surface due to the pulverization will continuously form a new SEI film, which leads to the decrease of charge and discharge efficiency and the acceleration of capacity attenuation [13].

In order to tackle the above-mentioned problems, the following strategies have been made in the past few years: (1) preparation of nano-sized silicon materials, such as the silicon nanoparticle [14,15], silicon nanowire [16,17], silicon nanotube [18,19] and silicon film [20]. The silicon nanomaterial provides large surface area and short ion diffusion path. The characteristics of high peristalsis and high plasticity of silicon nanomaterials can alleviate the volume effect at a certain extent and improve the cycling stability of the materials. Xinliang Feng et al. used block copolymers to construct independent two-dimensional structures model [21,22]. However, the existence and conservation of two-dimensional structure is very difficult; it is easy to agglomerate and form three-dimensional (3D) large particles. Therefore, the maintenance of two-dimensional structure is of great significance for the nanocrystallization of materials. (2) Preparation of silicon composites by introduction of the second phase, such as silicon/carbon composite material [23,24,25] and silicon-based metal complex [26,27]. The volume change of silicon during battery cycling is suppressed by the excellent mechanical properties of the second phase, and the conductivity of the composite can also be increased because of the introduction of the second phase. Thus, the rate performance of electrode will be improved. A combination of these two strategies is expected to be a promising approach toward high-performance silicon-based anode material. However, it is difficult to realize the controllable and large-scale synthesis of nanostructured silicon/carbon composite because low cost and scalable fabrication method free of using highly reactive silane species are rare [28,29]. Dong Jin Yoo et al. prepared a doped three-dimensional/small particle composite structure by reuniting with graphene oxide and platinum particles [30,31]. The composite has good catalytic activity and high stability in the field of fuel cell catalysts. Therefore, the three-dimensional structure and nano-size of materials are of great significance for improving the properties of materials. 

In this work, octavinyl-polyhedral oligomeric silsesquioxane is used as a silicon source and inorganic-organic crosslink agent for the synthesis of nano Si@C material. Because the polyhedral oligomeric silsesquioxane has a three-dimensional cage-like framework [32,33] and its size is at the nanometer level, the thus-developed Si@C negative electrode material prepared here preserves the three-dimensional pore structure of the silicon polyoxane. Such a unique morphology can alleviate the volume expansion of silicon material well. After magnesium heat reduction treatment, a conformable carbon coating is formed on the surface of silicon nanostructures. The coated carbon can also buffer the volume changes of silicon species during the cycling. At the same time, due to the existence of the pore structure in the developed Si@C material, the carbon phase in the composite would re-organize during the charge-discharge process, thus increasing the cycle life. Finally, the Si@C negative electrode material structure change along with the charge/discharge cycles of this synthesized material is investigated in order to get the mechanism of the capacity variation upon this kind of material during the whole life of charge-discharge cycles.

## 2. Materials and Methods 

Octavinyl-POSS(polyhedral oligomeric silsesquioxane), styrene and initiator AIBN (azobisisobutyronitrile) were purchased from Sigma-Aldrich Co. Ltd (Shanghai, China). Octavinyl-POSS, styrere and AIBN were added in a two-necked flask. After stirring for 10 min, the flask was placed in liquid nitrogen. During the freeze-pump-thaw cycles, oxygen was driven away. Then, the flask was transferred into the oil bath with mild magnetic stirring at 65 °C under nitrogen atmosphere. When the solution became transparent gel, copolymerization reaction finished. The production was vacuum dried at 80 °C for 9 h. After that, the poly(POSS-styrene) was carbonized at 900 °C for 3 h under the protection of Ar with a heating rate of 3 °C min^−1^. The as-obtained carbonized product was treated with magnesiothermic reduction at 650 °C for 2 h under the protection of Ar. The end-product was washed with 1 mol/L HCl and distilled water for several times and dried at 80 °C for 12 h. The Si@C anode material was then obtained. For comparison, the carbon material without Si was also prepared by etching the Si@C with analytic grade HF for 3 h, followed by repeated washing with ethanol six times.

The Si@C material was mixed with acetylene black and poly(vinylidene fluoride) (PVDF) to form a slurry at a mass ratio of 7:2:1 in N-Methyl pyrrolidone (NMP). After stirring for 12 h, the slurry was coated on copper foil by using the 90um spreader. Then, the slurry was dried at 70 °C for 12 h. The Si@C anode was then incorporated into coin cell in an argon filled glove box with a lithium foil as counter electrode, a Celgard separator and 1 M LiPF_6_ in a 1:1 ethyl carbonate (EC): dimethyl carbonate (DMC) solvent as electrolyte.

All the electrochemical tests were accomplished at room temperature. The galvanostatic charge-discharge tests were carried out using a Land CT2001A (whshland Co. Ltd., Wuhan, China) between 0.001–3 V. The cyclic voltammetry (CV) tests were carried out between 0.001–3 V by using a CHI660D electrochemical workstation (CH Instruments Co. Ltd., Shanghai, China) with a scan rate of 0.1 mVs^−1^. And the electrochemical impedance spectroscopy (EIS) tests were carried out between 0.001 Hz–10 KHz by using CorrTest CS310 electrochemical workstation (Wuhan Corrtest Instrument Co. Ltd., Wuhan, China) and the AC signal amplitude was 5 mV.

The material morphology and EDS mapping were observed by the scanning electron microscope (SEM, JSM-IT300) and the transmission electron microscope (TEM, Talos F200S, FEI, Waltham, MA, USA). The material structure was performed by the X-ray diffractometer (XRD, D8 Advance), the RENISHAW Raman microscope (Raman, RENISHAW InVia, Wotton-under-Edge, UK) and the X-ray photoelectron spectroscopy (XPS, ESCALAB 250Xi, Thermo Fisher Scientific, Waltham, MA, USA).

## 3. Results

**Figure 1:**
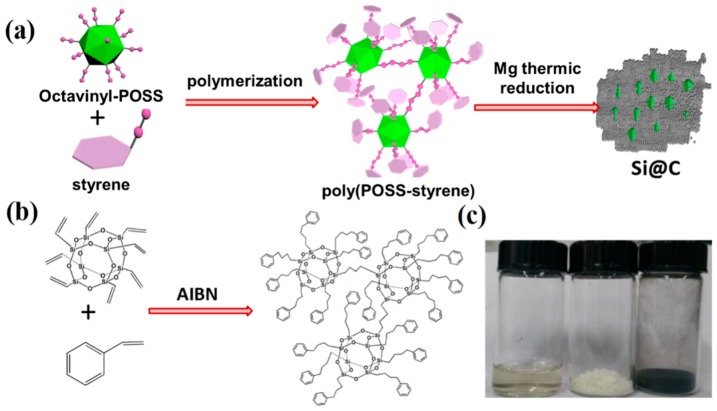
(**a**) Schematic illustration of synthesis of polyhedral oligomeric silsesquioxane (POSS)-derived Si@C anode material. (**b**) Formation of poly(POSS-styrene) between Octavinyl-POSS and styrene cross-linker via Vinyl copolymerization. (**c**) The photos of poly(POSS-styrene), dry-poly(POSS-styrene) and Si@C.

Figure 1a shows the synthesis process for the POSS-derived Si@C anode material. In this synthesis, octavinyl-POSS, as an organic-inorganic silicon source, introduces polyhedral silicon framework and nanoscale pores to the material system. Styrene is selected as a carbon source and a cross-linker to form 3D copolymer network as shown in Figure 1b. The formation of copolymer is based on copolymerization of vinyl monomers with a thermal initiator. After high temperature carbonization and magnesium reduction of the copolymer, carbon coated silicon species (Si@C) will be formed. The macroscopic morphology of the polymerized gel product and the final carbonized product are shown in Figure 1c.

**Figure 2:**
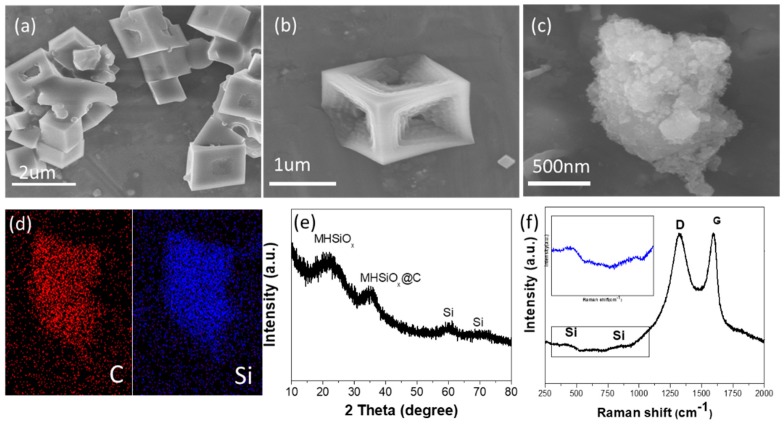
(**a**,**b**) Scanning electron microscope (SEM) images of poly(POSS-styrene) at different magnification. (**c**,**d**) SEM image and elemental mapping of Si@C. (**e**) X-ray diffractometer (XRD) patterns of Si@C. (**f**) Raman spectra of Si@C.

The poly (POSS-styrene) retains the 3D skeleton of octavinyl-POSS well, as shown in Figure 2a. The magnified SEM image of the poly(POSS-styrene) is shown in Figure 2b. After carbonization and reduction, the product exhibits a typical loose porous feature (Figure 2c). Element mapping of the final products reveals that carbon and silicon are evenly distributed in the sample (Figure 2d). Figure S1a,b (supplementary material) show XRD and Raman of POSS monomer and the polymer. The peak position of the polymer is roughly the same as the peak position of the monomer, indicating that the polymerization does not affect the crystal structure of POSS. 

Several broadening peaks are detected by the XRD measurement in Figure 2e, confirming the low degree of graphitization of carbon skeleton in Si@C. The XRD pattern shows that the magnesiothermic reduction process can promote the formation of Si@C composite structure. The broad peak around 25° can be attributed to MHSiO_2_ in amorphous, and another wide peak at 40° can be attributed to MHSiO_2_ complexed with amorphous carbon [34,35]. Raman in Figure 2f shows the broadening silicon peak at 400–500 cm^−1^ and 800–900 cm^−1^ [36]. In these Raman spectra, the D-band is located at 1340 cm^−1^ to characterize the defects of carbon atomic crystals and the G-band is located at 1556 cm^−1^ to characterize the plane expansion vibration of carbon atom SP2 hybrid [37,38]. The intensity ratio I_D_/I_G_ close to 1 further shows the low degree of graphitization of Si@C anode material.

**Figure 3:**
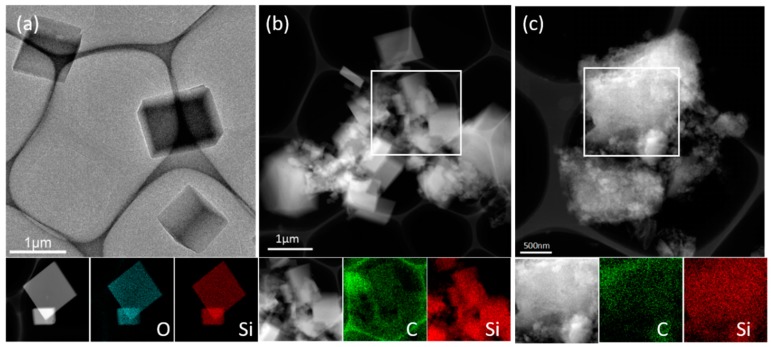
Transmission electron microscope (TEM) image and an area elemental mapping of poss (**a**), polymer (**b**) and Si@C (**c**).

The TEM image and elemental mapping of POSS monomer in Figure 3a show the octahedron morphology, confirm that the distribution of element in monomer is uniform. According to the area of elemental mapping of poly (POSS-styrene) in Figure 3b, carbon coats on the silicon surface after cross-linking and the integrity of the silicon skeleton well preserves. Figure 3c shows the morphology and elemental distribution of Si@C. It illustrates that even if the silicon skeleton collapses, silicon is still coated with carbon. This characteristic can help to buffer the expansion of silicon during charging and discharging.

**Figure 4:**
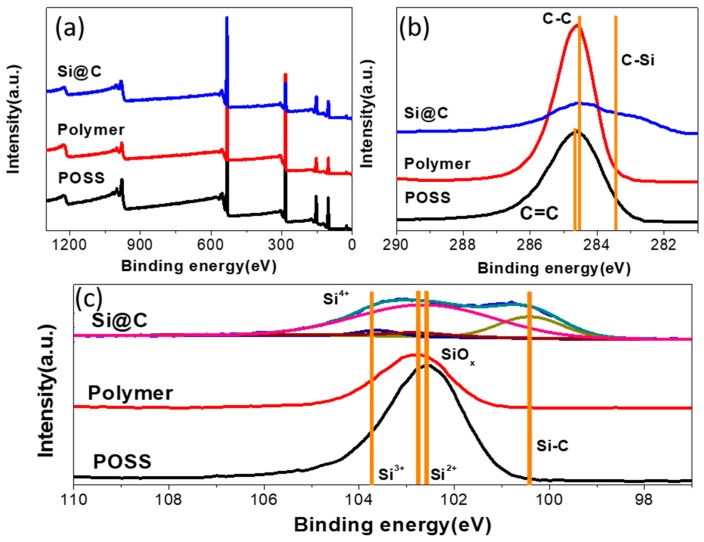
(**a**) X-ray photoelectron spectroscopy (XPS) survey spectra, and XPS spectra for the (**b**) C 1s and Si 2p (**c**) of POSS, polymer and Si@C.

The chemical states of POSS, Polymer and Si@C materials are analyzed by XPS. The accurate silicon content within the Si@C, determined by atomic absorption spectroscope, is 39.27 wt.%. As shown in Figure 4a, the full-range XPS surveys present the existence of silicon, carbon and oxygen. The high-resolution spectra of Si 2p and C 1s are shown in Figure 4b,c. After copolymerization, the peak position of SiOx located at 102.6 eV) shifts between POSS and polymer [39]. The Si 2p spectra of Si@C exhibits four peaks located at 100.4, 102.6, 102.8 and 103.6 eV, which correspond to Si-C, Si^2+^, Si^3+^ and Si^4+^, respectively. The presence of these Si species demonstrates the multiple oxidation states of Si in the composite and carbon phase is in close vicinity of Si species after carbonization and magnesiothermic reduction. In Figure 4b, to compare the spectra of C 1s between POSS and Polymer, the peak located at 284.5 and 284.6 eV corresponding to C=C and C-C indicates the copolymerization of vinyl radical. The spectra of C 1s of Si@C shows the peak of C-Si located at 283.5 eV, which is consistent with the peak of Si-C located at 100.4 eV.

**Figure 5:**
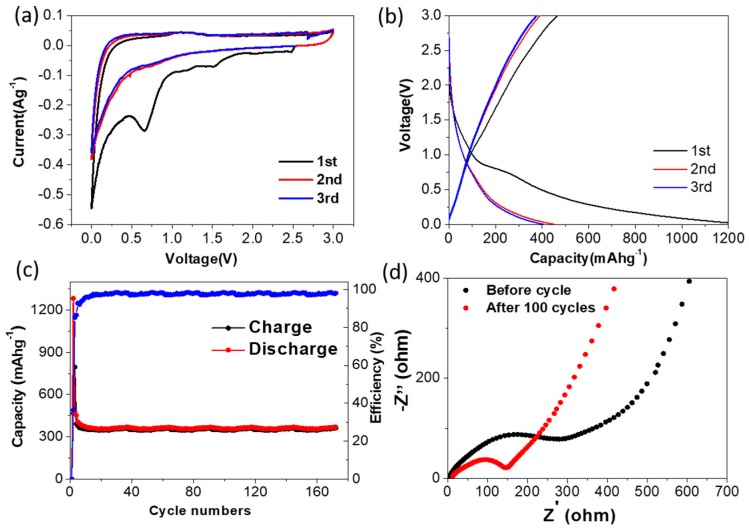
(**a**) First three CV curves of synthesized carbon material without Si at a scan rate of 0.1 mVs^−1^. (**b**) First three galvanostatic charge-discharge curves of synthesized carbon material without Si. (**c**) Cycling performance of synthesized carbon material without Si at a rate of 200 mAh g^−1^. (**d**) Electrochemical impedance spectra of synthesized carbon material without Si before and after cycling.

Electrochemical characteristics of synthesized carbon material without Si are shown in Figure 5. In the first three CV curves, as shown in Figure 5a, the redox peaks and potential plateaus are not obvious. As shown in Figure 5b, the capacity of the battery below 0.5 V was mainly contributed by lithium insertion into the graphitic layers. The appearance of a potential slope indicates the disordered stacking of the graphitic layers, which leads to electrochemically and geometrically nonequivalent active lithium sites [40,41]. The first cycle discharge capacity of synthesized carbon material without Si is about 1250 mAh g^−1^. After a couple of cycles, the discharge capacity attenuates drastically to 380mAh g^−1^ (in Figure 5b,c).

**Figure 6:**
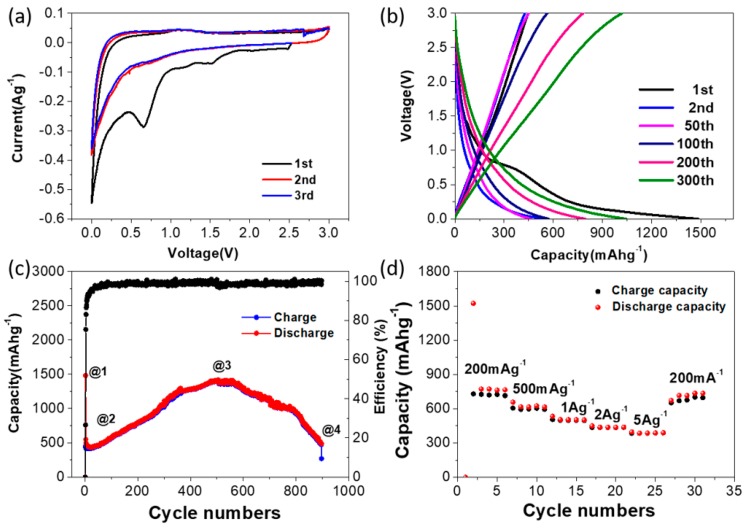
(**a**) First three CV curves of Si@C at a scan rate of 0.1mVs^−1^. (**b**) Galvanostatic charge-discharge curves of Si@C at a rate of 200mAg^−1^. (**c**) Full life cycling performance of Si@C at a rate of 200mAg^−1^. (**d**) Rate performance of Si@C at different rates.

The electrochemical characteristics of Si@C are shown in Figure 6. During the CV and charge-discharge curves (Figure 6a,b), the broad reduction peak (about 1.5 V) confirms the preliminary decomposition of the electrolyte and the formation of solid electrolyte interface (SEI) films. At about 1.3 V, the broad oxidation peaks are observed in the first three anodic scans, illustrating the reversible oxidation of some SEI components. At a charge-discharge rate of 200 mA g^−1^, the first cycle discharge and charge capacity of the Si@C electrode is 580 mAh g^−1^ and 1500Ahg^−1^, respectively as shown in Figure 6c. The large irreversible capacity exists here because of the formation of the solid electrolyte interphase (SEI) and some irreversible reactions upon the electrode and/or electrolyte [42]. There is an interesting phenomenon during charge-discharge process that the Si-C anode undergoes through a quick capacity drop-off at initial several cycles, and then the capacity creep up is observed along subsequent cycles; Finally, as the cycle continues, the capacity reaches the initial capacity (Figure 6c). Furthermore, the Si@C anode shows the superior rate performance as shown in Figure 6c. For instance, the Si@C anode presents relatively high reversible capacities of 570 mAh g^−1^, 500 mAh g^−1^ and 450 mAh g^−1^ at the rate of 1 A g^−1^, 2 A g^−1^ and 5 A g^−1^, respectively. When the current density goes back to 200 mA g^−1^, the reversible capacity can recover back to the initial value of 780 mAh g^−1^. The possible reason to the excellent rate performance of Si@C anode could be that its hollow cage structure offers a better electrode-electrolyte contact for the fast transmit of Li^+^ into Si@C material. In order to explore the reasons for the capacity change during the cycling, four representative analysis points were selected (number Si@C-@1, Si@C-@2, Si@C-@3 and Si@C-@4 shown in Figure 6c) and investigated for the mechanistic discussion. The Si@C-@1 point represents the fresh battery without cycling. The Si@C-@2 represents the battery after 20 cycles when the capacity reaches the lowest in the full cycle life. The Si@C-@3 represents the battery after 550 cycles when the capacity is the highest in the full cycle life. The Si@C-@4 represents the end point of the full cycle life. 

**Figure 7:**
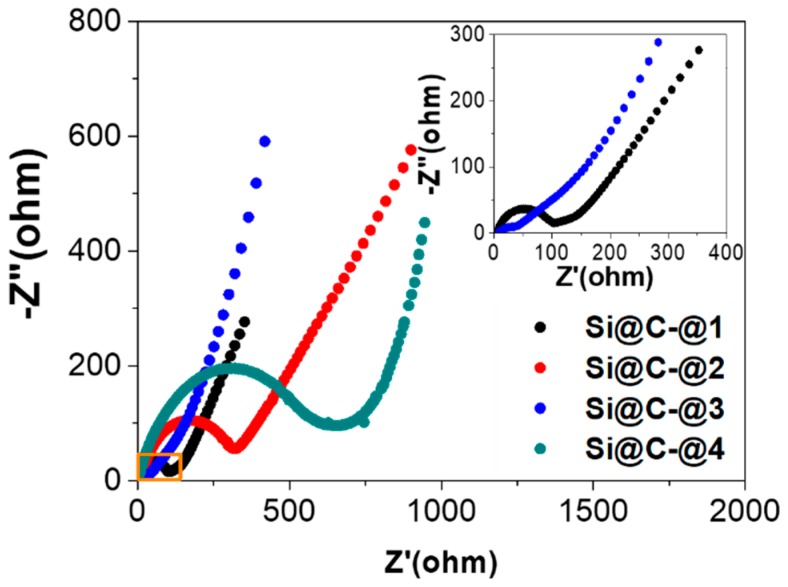
Electrochemical impedance spectra obtained at Si@C-@1, Si@C-@2, Si@C-@3 and Si@C-@4 points.

Electrochemical impedance measurements are carried out to discuss the electrode kinetic information of Si@C anode at these four analysis points (Figure 7). The Nyquist plot includes three parts: (1) a semicircle at high frequency region which characterizes partial de-solvation and adsorption of lithium ions onto the surface of the electrode; (2) a semicircle at intermediate frequency region representing the desolvated lithium ions into the lattice of active materials; (3) an oblique line at low frequency region relating to the solid-state diffusion of lithium ions [43,44,45]. In general, the electrode process is mainly controlled by the charge transfer and diffusion processes and the Nyquist diagram is composed of the semicircle in the high frequency region and the sloped line of the low frequency region. The resistance R_ct_ represents the charge transfer resistance of the electrochemical reaction and the charge transfer resistance has a great relationship with the electrochemical activity of the electrode material. The resistance R_ct_ of Si@C-@3 (~40 Ω) is minimum that may indicate the maximum activation of silicon and mutual embedment between silicon and carbon. The R_ct_ of Si@C-@2 (~300 Ω) is significantly larger than in the Si@C-@1 (~100 Ω) that illustrates the formation of solid electrolyte interphase (SEI) and the initial irreversible reaction during initial charge-discharge cycles. The resistance R_ct_ of Si@C-@4 (~500 Ω) is maximum that may indicate the structural collapse and the pulverization of grain after long-term charge-discharge. The obvious reduced resistance between Si@C-@2 and Si@C-@3 is beneficial for promoting the transport and storage of lithium ions, which explains the increased lithium storage capacity for as-obtained Si@C anode from 20 cycles to 550 cycles. The phenomenon of the increase on capacity after cycling had been reported for carbonaceous material. However, few reports explain the cause and mechanism of the rise of the capacity [46,47,48].

**Figure 8:**
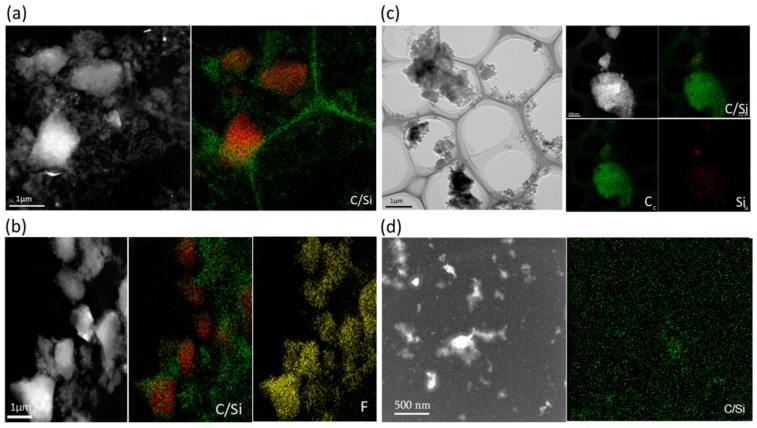
TEM image and elemental mapping of (**a**) Si@C-@1, (**b**) Si@C-@2, (**c**) Si@C-@3 and (**d**) Si@C-@4.

Furthermore, four representative cells cycled to the desired analysis points (in the complete delithiation condition) were disassembled in the glove box and Si@C anode material in these cells were characterized to further explain the mechanism for the capacity variation. Figure 8 shows the morphology and element distribution of the Si@C anode material in these four analysis points. Compared with Si@C-@1 (Figure 8), fluorine can be observed in Si@C-@2 (Figure 8). This result suggests that during the first charge and discharge process, the electrolyte diffuses into the sample to form a solid electrolyte membrane, causing an irreversible capacity change, which agrees with the rapid decline of the capacity at this time. In Si@C-@3 (Figure 8), the silicon surface is coated with a dense layer of carbon compared to Si@C-@1 and Si@C-@2. During the following cycles, carbon gradually penetrates into the silicon frame structure, relieves the volume expansion caused by the silicon itself in the process of charging and discharging and enhances the cyclic stability of the battery. At the same time, the continuous activation of silicon increases the lithium intercalation site and increases the lithium storage capacity, resulting in a capacity up to 1430 mAh g^−1^. In Figure 8, after long-term charge-discharge cycling, most of the carbon has been separated from the silicon surface and the structure collapsed, leading to a decline of capacity in later period.

**Figure 9:**
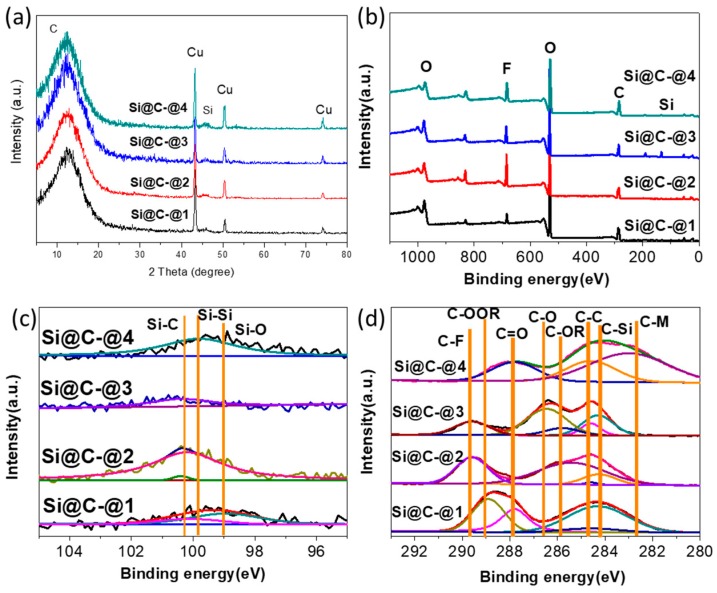
(**a**) XRD patterns of Si@C-@1, Si@C-@2, Si@C-@3 and Si@C-@4. (**b**) XPS survey spectra for Si@C-@1, Si@C-@2, Si@C-@3 and Si@C-@4. XPS spectra for the (**c**) Si 2p and (**d**) C 1s of Si@C-@1, Si@C-@2, Si@C-@3 and Si@C-@4. (M represents alkali metal or alkaline earth metal existed in the lithium salt and electrolyte additives.).

In Figure 9, by analyzing the composition of Si@C anode material in the four analysis points, the mechanism of capacity variation can be further unveiled. According to the small angle X-ray diffraction in Figure 9a, the peak of silicon (~46°) in Si@C-@4 is clearly observed due to the pulverization and abscission of silicon particles after a long-term charge and discharge. In the XPS survey spectra (Figure 9b), it is observed that the F 1s peak intensity in Si@C-@2 is the highest within four analysis points, indicating that the effect of the solid electrolyte membrane is dominant in Si@C-@2, which can explain the sharp capacity decline in the first few charge and discharge cycles. In Si 2p spectra (Figure 9c), compared to Si@C-@2, the chemical state of silicon in Si@C-@3 is mainly the chemical bond with carbon, indicates a conformable carbon coating on silicon, agreeing well the distribution of elements shown in Figure 9c. In Si@C-@4, the Si 2p spectra are dominated by silicon-silicon bonds. The similar trend can be also observed in XPS spectra for the C 1s (Figure 9d). Compared with Si@C-@3 and Si@C-@4, the intensity of Carbon-Fluorine peak is found to be the strongest in Si@C-@2. In Si@C-@4, the disappearance of the carbon-silicon peak matches well with the XPS spectra of the Si2p in the Si@C-@4 (Figure 9c). And long charge-discharge cycles, the electrolyte will decompose and volatilize to produce free HF, leading to insufficient electrolyte for complete charge-discharge reaction, thus resulting in the decline of the actual capacity. The appearance of free HF can damage the anode material and metal ion (Al, Mg, Ba, Li, Ca) in electrolyte can eliminate the free HF from the decomposition of LiPF_6_. The peak of the C-M appeared in Si@C-@4, indicating the destroying of the charge-discharge system.

## 4. Conclusions

In summary, POSS-derived Si@C was developed by carbonization of the precursor, followed by magnesium thermal reduction. As an LIBs anode material, it shows a good performance of lithium storage performance due to its unique nanostructure. The half battery with Si@C anode delivered an initial discharge capacity up to 1500 mAh g^−1^ and a high discharge capacity of 1430 mAh g^−1^ was retained after 550 cycles. In addition, we observed a rapid fade in discharge capacity of the electrode after 550 cycles. To understand such a discharge behavior, a combined analysis of the electrode with XRD, XPS, TEM and ex-situ impedance measurements were performed with Si@C anode at different cycling states. Our results show that the change in the discharge capacity is closely related to the changes of electrode composition induced by the electrolyte decomposition, agreeing with previous finds that the electrochemical properties of an electrode is closely related to the nanostructure of the material [49]. Our results demonstrate that the novel Si@C material is a promising alternative to existing graphite anode. In addition, the mechanism for the capacity change revealed in this study may provide useful guidance for future development of electrode materials. 

## Supplementary Materials

The following are available online at https://www.mdpi.com/2073-4360/11/4/576/s1, Figure S1: XRD and Raman spectra of POSS, Figure S2: electrochemical performance of the electrode.
