# Supplementary material for: POSS-Derived Synthesis and Full Life Structural Analysis of Si@C as Anode Material in Lithium Ion Battery
Source: Polymers (Basel). 2019 Mar 29;11(4):576. doi: 10.3390/polym11040576 (PMC6523519; doi:10.3390/polym11040576)
Supplement: Supplementary file 1 [file polymers-11-00576-s001.pdf]

Supporting Information

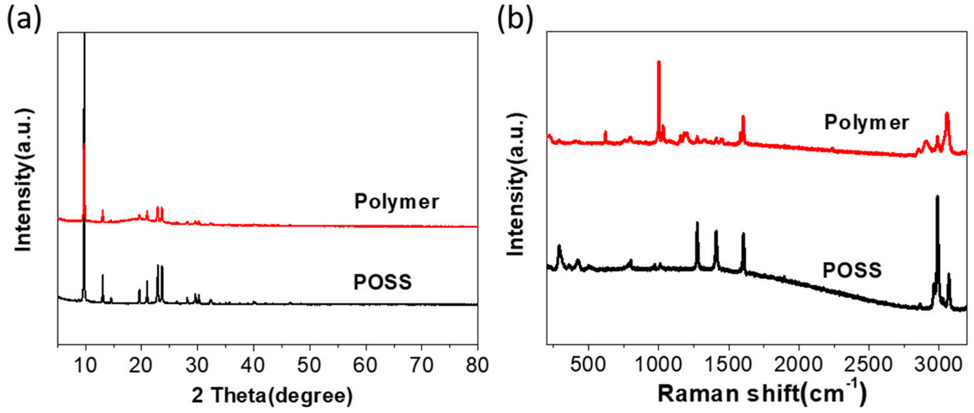

Figure.S1. (a) XRD patterns of POSS and Polymer. (b) Raman spectra of POSS and Polymer.
